# Camera Trapping: A Contemporary Approach to Monitoring Invasive Rodents in High Conservation Priority Ecosystems

**DOI:** 10.1371/journal.pone.0086592

**Published:** 2014-03-05

**Authors:** Anthony R. Rendall, Duncan R. Sutherland, Raylene Cooke, John White

**Affiliations:** 1 Centre for Integrative Ecology, School of Life and Environmental Sciences, Deakin University Melbourne, Victoria, Australia; 2 Research Department, Phillip Island Nature Parks, Cowes, Victoria, Australia; National University of Mongolia, Mongolia

## Abstract

Invasive rodent species have established on 80% of the world's islands causing significant damage to island environments. Insular ecosystems support proportionally more biodiversity than comparative mainland areas, highlighting them as critical for global biodiversity conservation. Few techniques currently exist to adequately detect, with high confidence, species that are trap-adverse such as the black rat, *Rattus rattus*, in high conservation priority areas where multiple non-target species persist. This study investigates the effectiveness of camera trapping for monitoring invasive rodents in high conservation areas, and the influence of habitat features and density of colonial-nesting seabirds on rodent relative activity levels to provide insights into their potential impacts. A total of 276 camera sites were established and left in situ for 8 days. Identified species were recorded in discrete 15 min intervals, referred to as ‘events’. In total, 19 804 events were recorded. From these, 31 species were identified comprising 25 native species and six introduced. Two introduced rodent species were detected: the black rat (90% of sites), and house mouse *Mus musculus* (56% of sites). Rodent activity of both black rats and house mice were positively associated with the structural density of habitats. Density of seabird burrows was not strongly associated with relative activity levels of rodents, yet rodents were still present in these areas. Camera trapping enabled a large number of rodents to be detected with confidence in site-specific absences and high resolution to quantify relative activity levels. This method enables detection of multiple species simultaneously with low impact (for both target and non-target individuals); an ideal strategy for monitoring trap-adverse invasive rodents in high conservation areas.

## Introduction

Invasive rodent species have established 80% of the world's island groups [1] causing significant damage to island environments [2]. In particular, four rodent species have frequently established on islands, the black rat (*Rattus rattus*), the brown rat (*Rattus norvegicus*), the Pacific rat (*Rattus exulans*), and the house mouse (*Mus musculus*) [3]–[4]. These rodents are opportunistic generalist feeders [5], highly adaptable to new environments, and as such are highly successful invaders [6]. Black rats are able to exploit a wide range of resources due to their arboreal nature [7] and are considered to exert the greatest impact of the three invasive rat species [8]. The impacts of mice on island systems are often less than those of rats [9]; however, alterations of native flora communities through seed predation and direct consumption by mice have been observed [10]–[11] as well as the reduction of invertebrate biomass on mouse invaded islands [12]. Mice have also been observed to prey upon seabirds in the absence of competitively dominant rats [13]–[14].

Invasive rodents on islands can significantly affect seabird populations [15]. Rodents have been implicated in 90% of avian extinctions on islands since 1600_AD_ [16] and are thought to be the most likely cause of extinction in 68% of Procellariiform seabirds [17]. Predation by rodents on seabirds occurs predominantly after brooding [18]–[19] when the parents go to sea, leaving their young undefended [20]–[22] resulting in reduced seabird breeding success [6].

Oceanic islands are an important source of global biodiversity [23]–[24], supporting increased levels of endemism compared with mainland regions [25]–[26]. The isolation of islands has resulted in the evolution of unique characteristics such as flightlessness in birds and, due to the absence of predators, fearlessness of mammalian predators [27]. Predators may be absent because they never colonised the island or because a predator population could not be sustained at some point in time [28]. It is this naivety of insular species to predators that makes them most vulnerable to invasive rodent predation [29].

The evidence for the impacts of invasive rodents on colonial seabirds is extensive (e.g [2], [17], [30]), however, monitoring techniques are often not suitable for use in areas where both invasive and native species coexist. Methods such as chew cards [31] and hair tunnels [32] have been found to be unreliable at certain densities and present the difficulty of confidently identifying species in diverse systems. Similarly snap traps pose an unacceptable risk to non-target species making their implementation unethical. Traditional live trapping methods can result in insufficient sample sizes [33] where trap adverse species, such as the black rat, are frequently under-detected [34]. Remote sensing cameras present a viable, low impact alternative to live trapping, and enable accurate identification of multiple species [35]. Using site occupancy of target species inferred from horizontally mounted cameras, De Bondi et al. [35] determined four surveying nights were required to obtain adequate confidence in black rat absences, compared to 215 live trap nights for the same level of confidence. This horizontal mounting technique was suggested to be two to five times more effective at determining medium-sized mammal presences when compared to the more commonly employed vertical mounting [36]. Despite some design inconsistencies including longer distances between camera and subject for horizontally mounted cameras, the authors attributed this result to the easier identification of body size, tail length, muzzle morphology and ear length [36]. Development of an effective and efficient method of surveying the often trap adverse invasive rodents is essential to further understand these species in high conservation value ecosystems.

This study therefore aims to:

1. Assess the detectability of invasive rodent species using horizontally mounted cameras,
2. To determine relative activity levels of invasive rodents in high conservation areas using horizontally mounted camera trapping,
3. To determine whether relative activity levels of rodents is associated with the density of colonial-nesting seabirds, the density of vegetation, the type of vegetation, or a combination of these.

## Methods

### Ethics Statement

Procedures carried out were in accordance with Deakin University Animal Ethics Committee approval (B12-2012), and the Department of Sustainability and Environment Wildlife permit 10006310.

### Study Site

The Summerland Peninsula, Phillip Island (145.13°E; 38.51°S) is located at the entrance of Western Port, southeast of Melbourne, Australia. The peninsula is about 360 hectares in size, surrounded by a rocky coastline and sandy beaches. Vegetation is dominated by blue tussock-grass (*Poa poiformis*), bower spinach (*Tetragonia implexicoma*), seaberry saltbush (*Rhagodia candolleana*), rounded noon-flower (*Disphyma crassifolium* subsp. *davellatum*) and coastal tea-tree (*Leptospermum laevigatum*) [37]. The peninsula is an area of high conservation significance supporting native species including little penguins (*Eudyptula minor*), short-tailed shearwaters (*Ardenna tenuirostris*), buff-banded rails (*Gallirallus philippensis*), hooded plovers (*Thinornis rubricollis*), and water rats (*Hydromys chrysogaster*).

### Site Selection

From April until late June 2012 50 cameras were rotated to monitor 276 camera-trap sites across the peninsula. Each camera was left in situ at each site for a minimum of eight nights. Sites were systematically established with an average density of one camera per 1.5 hectares, representing the identified home range size of black rats on Bagaud Island [38]. This spacing reduced the likelihood of detecting the same individual at multiple sites. A site was excluded if accessibility presented an unacceptable risk, and no site was positioned within 10 m of an active penguin burrow (short-tailed shearwaters were not present during the study). Active burrows were typified by either the presence of little penguins, the presence of recent diggings, excreta, or by penguin scent. Site locations were selected in the field, based on positions generated systematically, a priori, in ArcGIS [39].

### Camera Set-Up

**Figure 1:**
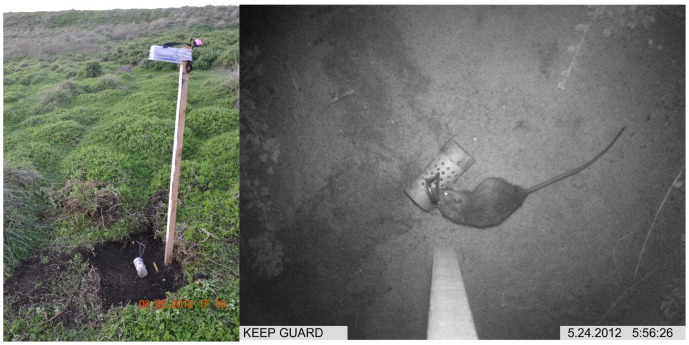
The set-up of the camera traps used in this research. Horizontal mounting setup demonstrating how the camera provides a ‘birds-eye view’ of the bait lure (left). Image of a black rat (right) demonstrating the type of images obtained, and the ease of identifying key features (tail length, ear morphology).

ScoutGuard 550 infrared motion-triggered cameras were mounted horizontally as first described in De Bondi et al. [35], providing a birds-eye view of the bait station (Figure 1). Cameras were mounted 1.3 m above the ground on wooden stakes. Approximately one square metre of vegetation was cleared at each camera site to reduce the incidence of false triggers caused by moving vegetation. This also enabled more accurate distinction of tail length, muzzle morphology and ear size of captured individuals, assisting with species identification. Black rats could be distinguished from native rats and house mice as they are greater than 100 mm in length with a distinctive tail that is longer than their head-body length with clearly visible large circular ears (Figure 1). A bait lure was secured to the ground below each camera site, ensuring the lure remained in place for the duration of the sampling period. Bait consisted of cotton wadding soaked in peanut butter, linseed oil, vanilla extract, and fish oil. This was secured within a heavily perforated polyvinyl chloride (PVC) tube 100 mm long. The standardised length of bait lures enabled accurate size measures of individuals to be determined, assisting in identification. Cameras remained in situ for eight nights and were set to take three consecutive photos (over a 7 s period) after each ‘trigger’ (motion under the camera). After each trigger the camera remained idle for 30 seconds to reduce the likelihood of re-sighting an individual on multiple occasions and to prevent memory cards (2 GB) filling up (∼1 400 images). Full memory cards at some sites resulted in fewer than eight nights' data being collected. To maximise detection of an animal, cameras were set on a ‘high’ sensitivity level.

### Index of Activity

To develop an index of activity for rodent species, each 24 h period of camera trapping was divided into 15 min intervals. Within each interval, the presence of a species was recorded. This interval was chosen as it provided a reasonable likelihood of only identifying an individual once given the bait was only an olfactory lure. The mean number of 15 min presences across each 24 h period represents the index of rodent activity at that site. This measure assumes that the level of activity is monotonic to the density of individuals within a given area. As such, areas of higher rodent activity are likely to represent greater densities. Such relationships between indices and abundance measures have been determined in other studies [32], [35]

### Habitat Assessments

Site specific habitat covariates were measured to determine the factors related to rodent activity levels. Three classes of factors were considered: seabird colony density as indicated by burrow density, floristic composition and the structural complexity of habitat. Colinearity was not present between covariates with all correlation coefficients having a value less than 0.55.

To develop a measure of burrow density, the point-centre quarter method [40] was used, measuring the distance to the nearest burrow in each of four directions (NE, NW, SE, SW) to a maximum distance of 40 m. Sites where no burrows were present were given a zero value. Burrows were identified as being either penguin or shearwater. Raw data for burrow density was analysed as described in Mitchell [40] using the formula:
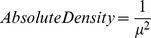
where *μ* is the mean distance to a burrow per site.

At each site, a 5 m×5 m quadrat was established, with the camera located at its centre. The percentage covers of the four most common floristic species found on the peninsula (*Poa poiformis*, *Tetragonia implexicoma*, *Rhagodia candolleana* and *Disphyma crassifolium*) were visually estimated. The remaining cover categorised as ‘other’. Structural complexity of habitats at each site was measured using a 1 m high structure pole [41] at eight points at each site, four in each corner of the quadrat, and four more 1 m either side of the camera. For each measurement, the number of touches of vegetation within each 100 mm interval (maximum ten touches) to a height of 1 m was determined. The more touches, the more complex the habitat. Vegetation cover above 1 m of the quadrat was visually estimated. Two categories representing meaningful intervals for rodent activity were developed for analysis, 100–400 mm representing near ground level complexity and >1 m reflecting upper story complexity.

### Statistical Analysis

The nightly probability of detecting target species with cameras was determined with the program PRESENCE version 4.9 [42]. The activity index was converted into a binomial presence/absence measure per night for this analysis. All species were modelled using constant nightly detection probabilities and non-constant detection probabilities, where an independent probability of detection is calculated for each survey night, both with constant occupancy. The cumulative nightly detection probability was determined using the formula:
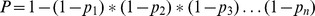
where *P* is the cumulative nightly detection probability, 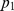 is the detection probability for night one, and *n* is the total number of nights cameras were active.

Generalised linear models (GLMs) were run in ‘R’ [43] using packages MASS [44] with model selection performed using package MuMIn [45]. A set of candidate models were developed a priori to explain rodent activity levels. These candidate models were chosen to represent plausible hypotheses of invasive rodent relative activity levels, influenced by the local density of colonial-nesting seabirds, floristic composition, structural complexity and relative activity levels of sympatric invasive rodents. Models were fitted with a negative binomial distribution underlying the counts of events per night (rodent activity index) as the data showed overdispersion. Akaike Information Criterion corrected for small sample sizes (AIC_c_) were used to select between models [46]. AIC_c_ weights (ω_i_) were calculated, giving the proportional weight for each model within the model set and model averaging was carried out where model selection uncertainty existed [46].

## Results

### Sampling Effort

There was a total of 2310 camera trap nights during the sampling period across 276 sites (mean = 8.4, median = 8.0, standard deviation = 1.7). In total, 186 501 images were taken with positively identified species assigned to 19 804 events (i.e. presences in 15 min periods). Throughout the study, 25 native species and six invasive species (black rat, house mouse, feral cat, European rabbit, brown hare, and common blackbird) were identified (Table 1). Black rats were most frequently detected with 11 261 events and were present at 90% of sites. House mice were detected and assigned to 3 220 events at 58% of sites.

**Table 1:** Number and percentage of sites at which species were detected through motion-triggered camera-trapping on the Summerland Peninsula, Phillip Island.

Common Name	Scientific Name	Sites Present	No. of Events*
**Eutherians**			
Black rat	*Rattus rattus*	248 (90%)	11 266
Brown hare	*Lepus cenchroides*	1 (0.4%)	2
European rabbit	*Oryctolagus cuniculus*	3 (1%)	4
Feral cat	*Felis catus*	25 (9%)	35
House mouse	*Mus musculus*	157 (57%)	3 222
Water rat	*Hydromys chrysogaster*	28 (10%)	50
**Marsupials**			
Common brushtail possum	*Trichosurus vulpecular*	157 (57%)	1 700
Common ringtail possum	*Pseudocheirus peregrines*	14 (5%)	26
Swamp wallaby	*Wallabia bicolor*	211 (76%)	2 394
**Monotremes**			
Short-beaked echidna	*Tachyglossus aculeatus*	51 (18%)	62
**Birds**			
Australian magpie	*Gymnorhina tibicen*	62 (22%)	168
Barn owl	*Tyto alba*	2 (0.7%)	2
Buff-banded rail	*Gallirallus philippensis*	2 (0.7%)	2
Cape barren geese	*Cereopsis novaehollandiae*	4 (1%)	6
Common blackbird	*Turdus merula*	4 (1%)	7
Grey shrike-thrush	*Colluricincla harmonica*	11 (4%)	17
Grey-currawong	*Strepera versicolour*	8 (3%)	11
Little penguin	*Eudyptula minor*	52 (19%)	409
Little raven	*Corvus mellori*	8 (3%)	15
Nankeen kestrel	*Falco cenchroides*	1 (0.4%)	1
Pied currawong	*Strepera graculina*	1 (0.4%)	1
Purple swamphen	*Porphyrio porphyrio*	6 (2%)	17
Red-browed finch	*Neochmia temporalis*	1 (0.4%)	1
Short-tailed shearwater	*Ardenna tenuirostris*	2 (0.7%)	2
Singing honeyeater	*Lichenostomus virescens*	2 (0.7%)	2
Superb fairy wren	*Malurus cyaneus*	45 (16%)	192
White-browed scrubwren	*Sericornis frontalis*	22 (8%)	81
White-faced heron	*Pelagodroma marina*	1 (0.4%)	1
White-fronted chat	*Epthianura albifrons*	4 (1%)	5
Willie-wag tail	*Rhipidura leucophyrys*	17 (6%)	33
**Reptiles**			
Blotched blue-tongue lizard	*Tiliqua nigrolutea*	2 (0.7%)	3
Unknown skink		17 (6%)	54

*An event is considered when a species is identified within a 15 minute time period. A single event may represent multiple individual triggers.

### Detection Probability

**Figure 2:**
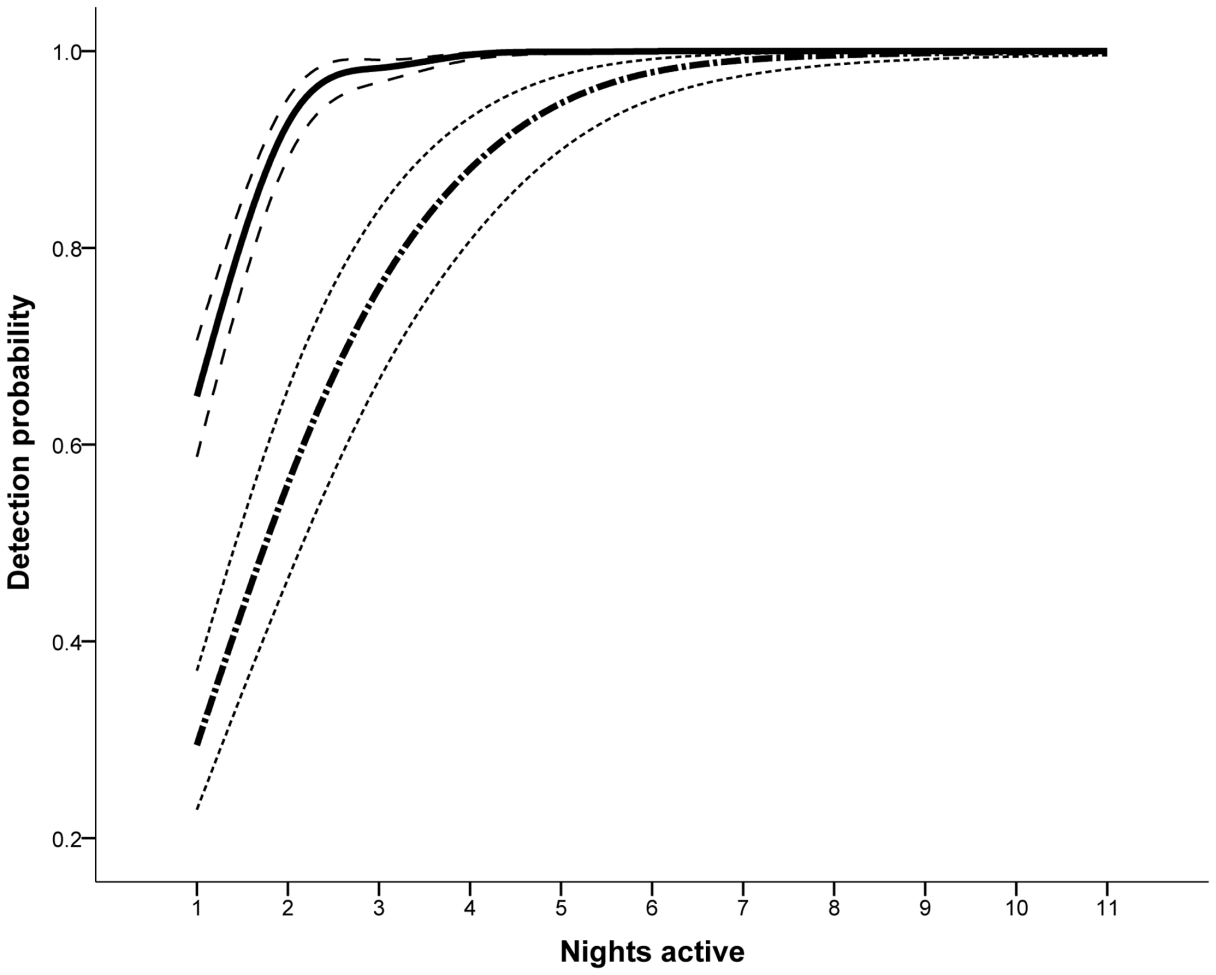
The detection probabilities obtained using this camera trapping approach. Nightly detection probability, including upper and lower 95% confidence intervals for black rats (solid line) and house mice (dot-dashed line).

Site level detection probabilities were calculated for both black rats and house mice. Black rats had an estimated occupancy of 90% across all sites, compared to house mice with an estimated 58% across all sites. Models were run with a constant nightly detection probability, with constant occupancy; and a non-constant nightly detection probability, with constant occupancy for each species. Both black rats and house mice were found to have a non-constant nightly detection probability with these models receiving full support (ω>0.95) as the best model in both cases (Table 2). Black rats had a high nightly detection probability ranging from 0.30 to 0.79 resulting in only three camera trap nights being required to be 95% confident of site-level absence (Figure 2). House mice had a comparatively lower nightly detection probability ranging from 0.29 to 0.59 resulting in just five nights being required to obtain the same level of confidence (Figure 2).

**Table 2:** AICc model selection for the detection probabilities for two species with the potential to impact on the conservation values of the Summerland Peninsula.

Species	Model*	K	AICc	ΔAIC	ω_i_	Log Likelihood (−2)
**Black rat**	psi(.).p(night)	12	2601.08	0.00	1.00	2577.08
	psi(.).p(.)	2	2631.84	30.76	0.00	2627.84
**House mouse**	psi(.).p(night)	12	2215.64	0.00	1.00	2191.64
	psi(.).p(.)	2	2243.78	28.14	0.00	2239.78

*Model variables include: psi(.).p(.) = constant occupancy across sites and constant nightly detection probability; psi(.).(night) = constant occupancy across sites and temporal effect on nightly detection probabilities.

Values represent the number of parameters (K), Akaike Information Criterion, corrected (AICc), AICc differences (ΔAICc), Akaike weights (ω_i_) and Log likelihood.

### Activity Index

**Figure 3:**
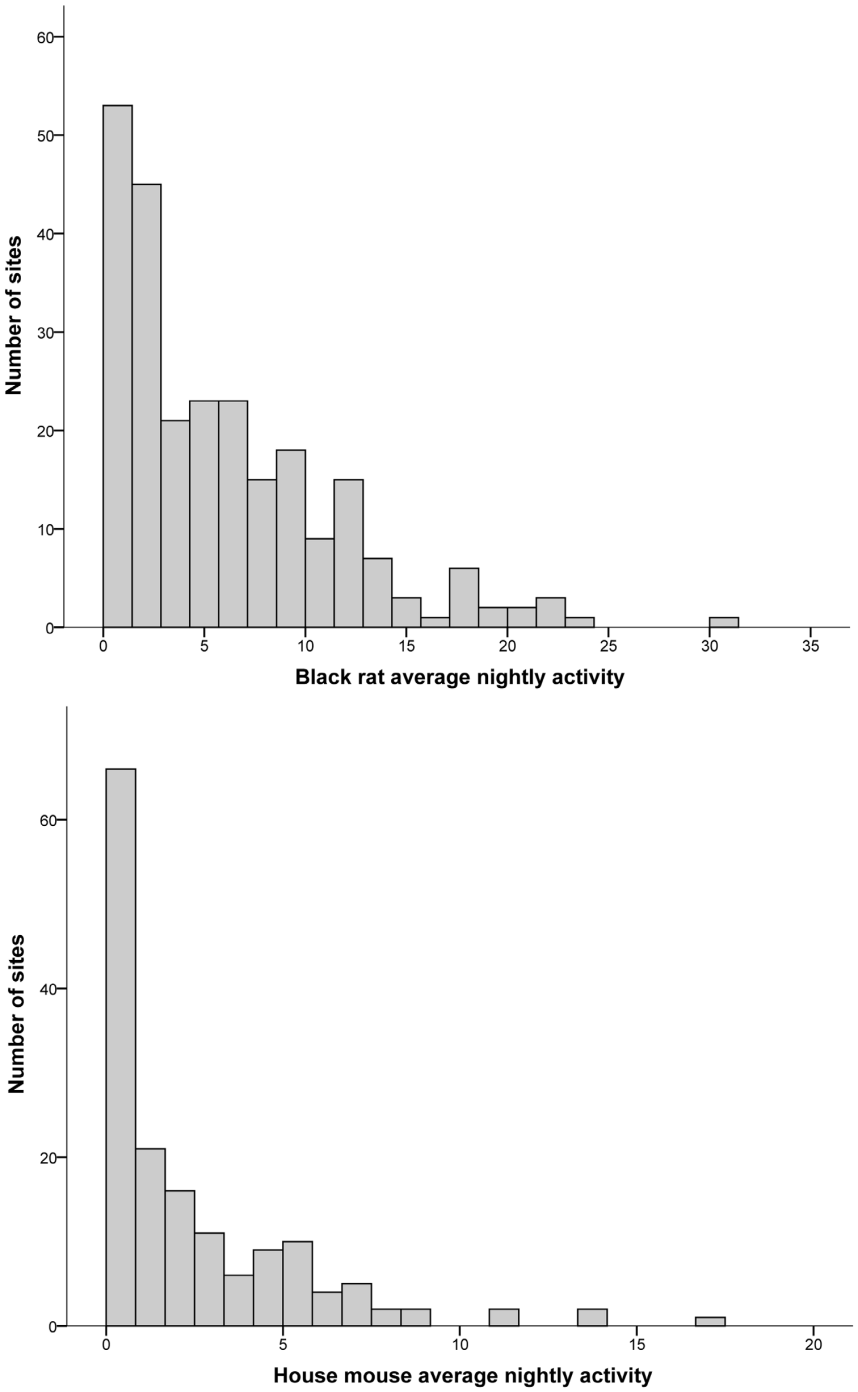
Histogram demonstrating the variation levels in the activity index derived from camera trapping. The number of sites at which activity index measures (mean number of events per night) were observed for black rats (a) and house mice (b).

For an activity measure to adequately assess habitat correlates adequate variation in activity levels is required. To assess this, the mean numbers of events per night were plotted against the frequency of their occurrence (Figure 3). This demonstrates the variation of the index measure, with black rat activity ranging from an average of one event per night up to an average of 31 events per night (mean = 5.4, median = 3.8). House mice, similarly, ranged from an average of one event per night, to an average of 17 events per night (mean = 1.4, median = 0.125) (Figure 3).

### Black Rats

**Figure 4:**
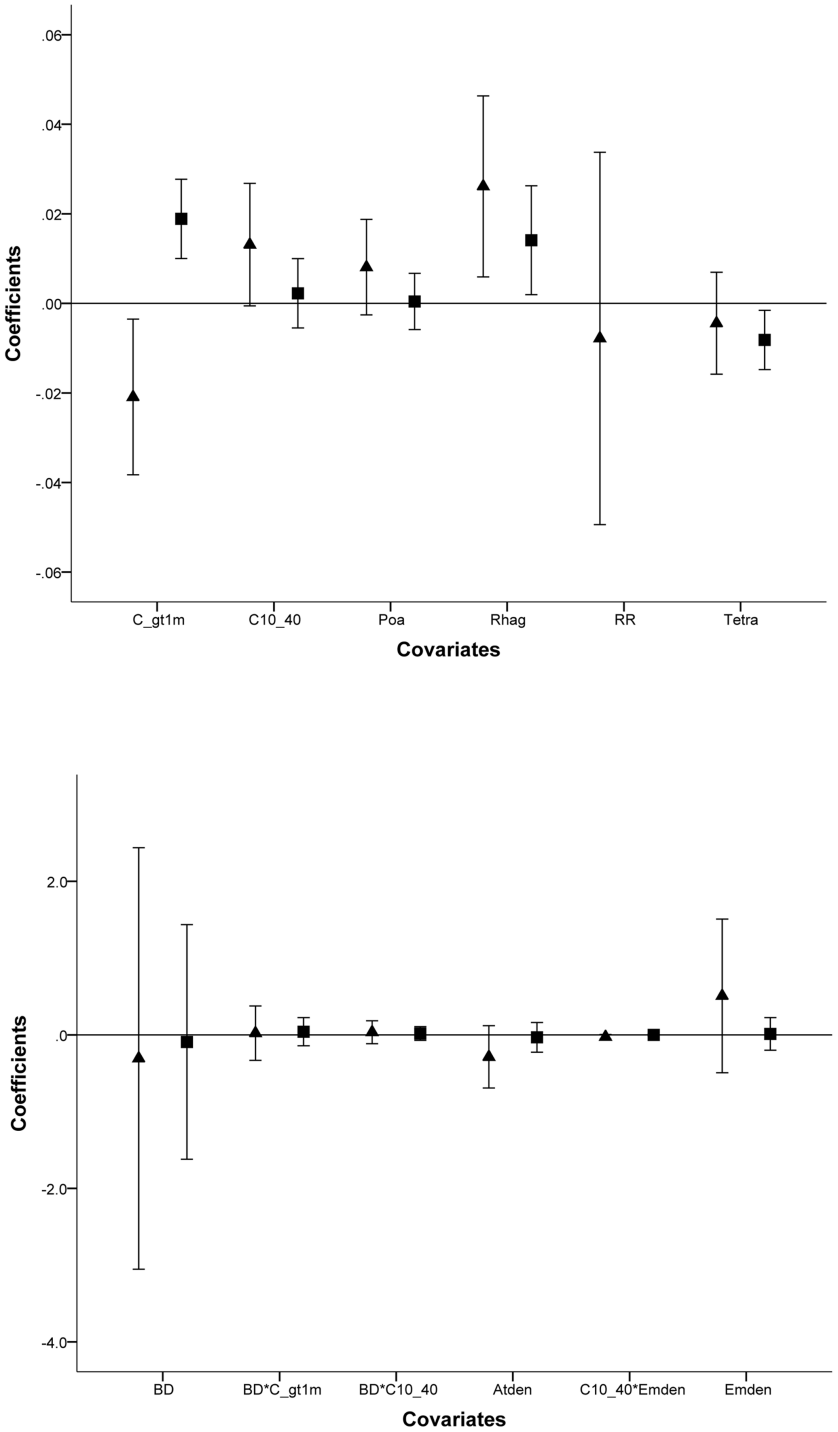
Error bar plot indicating the influence of model covariates on rodent activity levels. Model averaged coefficients (mean±95% CI) for black rats (square) and house mice (triangle) demonstrating their influence on activity levels for floristic and structural covariates (a) and covariates including burrow density (b).

The black rat was the most detected species across the peninsula. The models (Table 3) were run with an underlying negative binomial distribution. Structural complexity greater than 1 m was supported as the most influential covariate, being in the top model (Table 3) and had a positive influence on black rat relative activity levels; whereas seabird burrow density and vegetation structural complexity between 100–400 mm had little influence (Figure 4). Models describing vegetation floristic composition as a driver of black rat activity were not supported.

**Table 3:** AICc based model selection for different species identified through camera trapping on the Summerland Peninsula.

	Model	df	AICc	ΔAIC	ω_i_
**Black**	C_gt1m	3	1522.1	0.00	0.665
**rat**	C_gt1m+BD	4	1524.1	2.06	0.238
	C_gt1m+BD+C_gt1m*BD	5	1526.0	3.95	0.092
	Poa+Tetra+Rhag	5	1532.3	10.19	0.004
	Constant	2	1536.4	14.30	0.001
	C10_40	3	1537.9	15.86	0.000
	BD	3	1538.1	16.04	0.000
	Atden	3	1538.3	16.26	0.000
	Emden	3	1538.4	16.32	0.000
	C10_40+BD	4	1539.7	17.59	0.000
	C10_40+BD+C10_40*BD	5	1541.6	19.52	0.000
	Emden+C10_40+Emden*C10_40	5	1542.1	19.99	0.000
**House**	C_gt1m	3	846.9	0.00	0.297
**mouse**	Poa+Tetra+Rhag	5	847.6	0.72	0.207
	C_gt1m+BD	4	848.9	2.00	0.109
	C10_40	3	849.7	2.77	0.075
	Constant	2	850.0	3.07	0.064
	Atden	3	850.2	3.31	0.057
	Emden+C10_40+Emden*C10_40	5	850.9	3.97	0.041
	C_gt1m+BD+C_gt1m*BD	5	851.0	4.06	0.039
	C10_40+BD	4	851.7	4.81	0.027
	Emden	3	851.8	4.87	0.026
	RR	3	851.9	5.00	0.024
	BD	3	852.0	5.11	0.023
	C10_40+BD+C10_40*BD	5	853.6	6.71	0.010

Model covariates include: Vegetation cover above 1 metre (C_gt1m), vegetation cover between 10 and 40 cm (C10_40), percentage cover of *Poa poiformis* (Poa), percentage cover of *Tetragonia implexicoma* (Tetra), percentage cover of *Rhagodia candolleana* (Rhag), short-tailed shearwater burrow density (Atden), little penguin burrow density (Emden), Atden and Emden combined (BD), and black rat activity (RR).

Values represent the number of parameters (df), Akaike Information Criterion, corrected (AICc), AICc differences (ΔAICc), Akaike weights (ω_i_).

### House Mouse

House mice were the second most detected species on the peninsula. Competing models were run with an underlying negative binomial distribution (Table 3). There is model selection uncertainty as indicated by the ω_i_ of the two most parsimonious models (Table 3). Structural complexity greater than 1 m had a negative influence on mouse activity levels whereas greater seaberry saltbush cover and greater structural complexity between 100–400 mm had a positive influence (Figure 4). Seabird burrow density was not informative and showed little association with mouse activity (Table 3). Similarly black rat activity levels did not influence mouse activity (Table 3) In contrast to black rats, house mice were influenced more by vegetation species composition with these models receiving considerable support (Table 3).

## Discussion

The use of remote sensing cameras in environmental research is increasing, with studies now demonstrating cameras can be more effective than alternative index measures when monitoring terrestrial mammals [47]. This study has demonstrated that remote sensing cameras represent an effective and efficient alternative to live trapping techniques in areas where non-target species are present and detection rates for trapping target species are low. Multiple detections of invasive rodents within and between nights using horizontally mounted cameras allowed relative activity measures to be calculated with high precision. The ability to rapidly establish relative activity levels with high confidence provides an ideal platform to assess the correlates of invasive rodent activity with minimal impact on target and non-target species. Detection of multiple species through a singular method enables a more holistic understanding of species communities enabling simultaneous monitoring of these species. Furthermore, methods of monitoring invasive species without impacting non-target species are highly desirable. Remote-sensing cameras provide a viable method for surveying areas of high conservation significance whilst negating target and non-target impacts of live trapping and animal welfare concerns of using wildlife for research purposes [48]–[50].

The key correlate of black rat activity was structural complexity of vegetation more than one metre above ground, predominantly tree cover, which has been observed in other studies [51]–[53], and is believed to be a strategy of avian predator avoidance [29], [54]. These areas of greater structural complexity also may provide higher resource availability for the more arboreal black rats [55]. The strong association of black rat activity and structural complexity greater than 1 m implies there could be considerable potential for negative interactions between black rats and woodland bird species. Black rats are known to predate on tree-nesting birds [1], [56], and for example have caused the extinction of five endemic woodland birds on Lord Howe Island [1]. In contrast, house mouse activity was associated with lower structural complexity between 100–400 mm, similarly providing protection from avian predation as shown in experimental studies [57]–[58]. The resource partitioning suggested between black rats and house mice may reflect the dominance of black rats resulting in the subordinate house mouse altering its activities to occupy an alternative niche [59]–[61]. Despite the potential dominance of black rats over house mice there was considerable overlap between their ranges. This suggests that although black rats may outcompete house mice for certain resources, they do not actively exclude them from these areas as suggested with black rat activity not being an influential covariate for house mouse activity, this has also been shown in other studies [51], [61].

Lower rodent detection was observed in less structurally complex habitats such as core areas of seabird breeding colonies. Increasing levels of habitat complexity were found at the peripheries of colonies. The association of rodent activity with structural complexity highlights these peripheral regions as likely to experience intensified interactions with invasive rodents. The physical disturbance of seabirds on islands reduces the overall structural complexity of habitats within colonies [61] and through such activities may be reducing the access of these areas to rodents. Irrespective of this, black rats and house mice were still present throughout colonial seabird colonies. Results did not suggest a negative relationship of rodents to burrow density, therefore it is unlikely seabirds are excluding rodents.

### Future Research

The broad distribution of black rats in conjunction with their extreme activity levels in certain regions has serious implications for wildlife management. The potential impacts of black rats could be significant given the evidence that has been recorded for exotic rodents within island ecosystems [1], [3], [5]. Investigation of the impacts of black rats on colonial seabird and woodland bird communities in Australia is needed. Confirming whether black rats prey upon seabird eggs or chicks on Phillip Island through dietary investigations and direct observations of predation events, using remote sensing cameras, is a priority. Predation on juvenile seabirds has been identified as the most likely cause of population decline in many seabird species [16], [62]–[63]. Investigating breeding success rates of little penguins and short-tailed shearwaters in relation to a gradient of structural complexity is warranted to identify whether breeding failure rates are exacerbated by higher predation in dense vegetation.

This study demonstrates considerable data on multiple species is obtainable, with high confidence in absences, enabling inferences to be made from an index measure, without the need of site-specific density measures. Comparison between activity index measures and population density measures through mark-recapture studies could reveal the efficacy of remote sensing cameras. The ability to estimate density without identifying individuals is an area for future research, with analysis on smaller count data sets showing promise e.g. [64]–[66]. The ability to confidently determine absences could have significant application in invasive species eradication with this method representing a low impact method for analysing the efficacy of such campaigns, particularly where non-target species are present. Similarly, the ability to collect data on multiple species simultaneously (Table 1) could significantly reduce survey effort in these regions, reducing overall impacts and increasing survey efficacy.
